# Fuzzy ensemble of fined tuned BERT models for domain-specific sentiment analysis of software engineering dataset

**DOI:** 10.1371/journal.pone.0300279

**Published:** 2024-05-28

**Authors:** Zeeshan Anwar, Hammad Afzal, Naima Altaf, Seifedine Kadry, Jungeun Kim

**Affiliations:** 1 Department of Computer Software Engineering, National University of Sciences and Technology, Islamabad, Pakistan; 2 Department of Applied Data Science, Noroff University College, Kristiansand, Norway; 3 Artificial Intelligence Research Center (AIRC), Ajman University, Ajman, United Arab Emirates; 4 Department of Electrical and Computer Engineering, Lebanese American University, Byblos, Lebanon; 5 MEU Research Unit, Middle East University, Amman, Jordan; 6 Dept. of Software, Kongju National University, Gongju-si, Korea; Zayed University, UNITED ARAB EMIRATES

## Abstract

Software engineers post their opinions about various topics on social media that can be collectively mined using Sentiment Analysis. Analyzing this opinion is useful because it can provide insight into developers’ feedback about various tools and topics. General-purpose sentiment analysis tools do not work well in the software domain because most of these tools are trained on movies and review datasets. Therefore, efforts are underway to develop domain-specific sentiment analysis tools for the Software Engineering (SE) domain. However, existing domain-specific tools for SE struggle to compute negative and neutral sentiments and can not be used on all SE datasets. This work uses a hybrid technique based on deep learning and a fine-tuned BERT model, i.e., Bert-Base, Bert-Large, Bert-LSTM, Bert-GRU, and Bert-CNN presented that is adapted as a domain-specific sentiment analysis tool for Community Question Answering datasets (named as Fuzzy Ensemble). Five different variants of fine-tuned BERT on the SE dataset are developed, and an ensemble of these fine-tuned models is taken using fuzzy logic. The trained model is evaluated on four publicly available benchmark datasets, i.e., Stack Overflow, JavaLib, Jira, and Code Review, using various evaluation metrics. The fuzzy Ensemble model is also compared with the state-of-the-art sentiment analysis tools for the software engineering domain, i.e., SentiStrength-SE, Senti4SD, SentiCR, and Generative Pre-Training Transformer (GPT). GPT mode is fine-tuned by the authors for domain-specific sentiment analysis. The Fuzzy Ensemble model covers the limitation of existing tools and improve accuracy to predict neutral sentiments even on diverse dataset. The fuzzy Ensemble model performs superior to state-of-the-art tools by achieving a maximum F1-score of 0.883.

## 1 Introduction

Opinion mining or sentiment analysis is a technique to mine the emotion/ sentiment of users from textual data [1]. Sentiment analysis can be applied on document level, sentence or phrase level, or aspect level [2]. The accuracy of sentiment analysis often depends on the lexicon/dictionary. The current research shows that the domain-specific lexicons often outperform the generic lexicon in terms of precision and accuracy [3].

Like other domains, sentiment analysis of software engineering text is valuable [4] and has various applications such as getting the opinion of users [5], analyzing the quality of content [6], analyzing the product review [7] and identifying community smells [8]. There are various sources to extract software engineering text some of the popular sources include community question-answering sites (Stack Overflow, Quora, Yahoo Answers, etc.), bug reports, and app reviews. Certain studies have been performed for domain-specific sentiment analysis of software engineering text, and tools are also developed for this purpose. For example, Obadi et al. [9] performed a systematic mapping study of sentiment analysis tools developed for software engineering. Lin et al. [10] observed that existing sentiment analysis tools are not suitable for the SE domain and produce negative and biased results.

Sentiment analysis in the domain of software engineering has gained significant attention due to its relevance in understanding the sentiments expressed in various software-related interactions. However, existing domain-specific sentiment analysis tools in software engineering have certain limitations, prompting the need for an in-depth analysis and the development of improved tools. The aim of this research is to address the following research questions:

**RQ1:** What are the limitations of existing domain-specific sentiment analysis tools for software engineering?
  This question directs our exploration into the challenges and shortcomings of currently available sentiment analysis tools tailored for software engineering domain.
**RQ2:** How to design a domain-specific tool to overcome the limitations of existing sentiment analysis tools for software engineering?
  This question focuses on the methodology and strategies employed to develop a sentiment analysis tool specifically tailored to the software engineering language and interactions.
**RQ3:** To what extent does the proposed tool overcome the identified limitations?
  This question forms the core of our evaluation, aiming to quantify and qualify the effectiveness of the newly proposed sentiment analysis tool in mitigating the limitations identified in existing tools.

The limitations of existing tools for sentiment analysis were identified after an extensive review of the literature. These limitations answer the **RQ1** and can be enlisted as i) struggle to compute negative and neutral [11] sentiments on SE text; ii) only work well on a particular dataset on which these are trained and iii) are not suitable as general-purpose resource [12] as results are often biased. Authors also studied and experimented with various machine learning techniques [13–19] for sentiment analysis, but the results are quite diverse.

To overcome these limitations, five variants of BERT are developed, fine-tuned, and trained on SentiSE [20] dataset to compute the sentiment of SE text. The results of BERT models i.e. Bert-Base, Bert-Large, Bert-LSTM, Bert-GRU, and Bert-CNN are ensembles using fuzzy logic to compute the sentiment from SE text. The effectiveness of the fuzzy ensemble model is evaluated on four benchmark datasets. The results are also compared with the existing techniques, i.e., SentiStrength-SE, Senti4SD, SentiCR, and GPT. From the evaluation of results, it is concluded that newly proposed Fuzzy Ensemble model gives the best results on all the datasets. The results indicate that both individual BERT models and the proposed fuzzy ensemble model demonstrate notable performance in predicting neutral and positive sentiment classes. This achievement addresses a common limitation observed in existing models that often struggle with accurate predictions for neutral sentiment. The model is trained on one dataset and tested on various datasets. The performance of the proposed fuzzy ensemble is not only comparable with the existing tools but it is better than the existing tools on Stack Overflow, Jira, and CodeReview datasets. The incorporation of a fuzzy ensemble mechanism provides a holistic approach to sentiment analysis, effectively leveraging the strengths of multiple BERT models. The comparison of accuracy rates for positive, negative, and neutral classes shows performance across different sentiment categories. These findings collectively underscore the efficacy of the ensemble approach in achieving robust sentiment prediction.

The major contributions of this article are enlisted below:

- We contribute by designing and fine-tuning various BERT-based models specifically tailored for sentiment analysis in the software engineering domain. Additionally, we introduce a novel fuzzy ensemble model that combines the strengths of these fine-tuned BERT models through fuzzy logic mechanisms and overcome the limitations of exiting models. This ensemble approach enhances the accuracy and interpretability of sentiment predictions in the software engineering text.
- A distinctive contribution of our work is the fine-tuning of the Generative Pre-Training Transformer (GPT) specifically for sentiment analysis tasks within the software engineering domain. To the best of our knowledge, this is the first instance of fine-tuning GPT for sentiment analysis in the software engineering domain.
- We conduct an extensive and comprehensive study, evaluating the performance of our developed models across four benchmark datasets within the software engineering domain. Furthermore, we benchmark our models against existing state-of-the-art tools commonly used in sentiment analysis tasks related to software engineering. This large-scale evaluation provides valuable insights into the efficacy and versatility of our models in comparison to existing tools.
- Our fine-tuned BERT-based fuzzy ensemble model achieves a notable F1-score of 0.88 on the Stack Overflow dataset. This result surpasses the performance of all existing BERT-based models in sentiment analysis within the software engineering domain. The achievement of a high F1-score signifies the superior precision and recall of our model.

The organization of remaining sections of this article is as follows. A review of related work is made in Section 2. Research methodology is explained in Section 3. Experiments are given in Section 4 and the Discussion Section 5 include the Evaluation of the proposed model with published dataset is made in Section 5.1. Furthermore, the proposed methods is compared with state-of-the-art sentiment analysis tools in Section 5.2. The consequences of fine-tuning LLMs and application of the proposed sentiment analysis model is also included in the Discussion Section 5. Finally, Section 6 concludes the paper.

## 2 Related work

General-purpose sentiment analysis tools do not perform well in the SE domain because these tools are not created by using specialized vocabulary. Few tools for the SE domain are created but these tools are tested against the general-purpose tools. The purpose of Islam et al. [21] study is to investigate which tool is best for the SE domain and to what extent predictions of these tools agree/disagree with each other. Stack Overflow posts, JIRA issue comments, and Code review comments authors created datasets, and three sentiment analysis tools named SentiStrength-SE, Senti4SD, and EmoTxt were tested. SentiStrength-SE achieved the highest accuracy on the JIRA issue comments, whereas, Senti4SD had high accuracy on the Stack Overflow dataset. The overall average accuracy of SentiStrength-SE is 65.26% on the Code review dataset. It is observed that all these tools struggle to compute negative sentiments.

In the SE domain, sentiment classifiers trained on general-purpose datasets are biased in classifying neutral sentences as negative. A balanced Gold standard dataset consisting of 4423 posts was developed manually. Distributed Semantic model was created by using 20 million Stack Overflow posts. The authors tried to overcome this by creating an SVM classifier using lexicon-based, keyword-based, and semantic features using the R interface. 25x features ranked by information gain were selected. Senti4SD was compared with SentiStrength and SentiStrength-SE. Calefato et al. [22] observe 19% improvements in precision for negative and 25% improvements in recall for the neutral class compared with SentiStrength.

In another research, Lin et al. [10] developed a sentiment analysis technique for a software library recommender system. Authors extracted 40k sentences from stack overflow and manually labeled them positive, neutral, and negative. Recursive Neural Network (RNN) was used to compute the sentiment of sentences. The newly developed technique is named Stanford CoreNLP–SO. The proposed technique was applied to three SE datasets: Stack Overflow, Mobile app reviews, and JIRA issue comments. The technique was compared with four cutting-edge techniques named SentiStrength, NLTK, Stanford CoreNLP, and SentiStrength–SE. Precision and recall are used as evaluation metrics. After extensive training, the authors observed the negative results. The results of all the techniques are comparable but not satisfactory to recommend the APIs. It concludes that sentiment analysis for SE is not yet mature and requires the attention of researchers.

Novielli et al. did a study to replicate the results of [21]. The authors include all three cutting-edge tools available for sentiment analysis of the SE domain. This study investigates which tool is best for the SE domain and to what extent these tools agree/disagree with each other. JIRA issue comments, Stack Overflow posts, Code review comments, and Java libraries authors created datasets, and three sentiment analysis tools named SentiStrength-SE, Senti4SD, and SentiCR were tested. The accuracy of tools is detected by measuring the precision, recall, and F-score. SE-specific tools performed well as compared with baseline SentiStrength. SentiStrength-SE achieved the highest accuracy on the JIRA dataset whereas, Senti4SD had high accuracy on the Stack Overflow dataset. The F-measure for Senti4SD is 0.84 on SO data [12].

Islam et al. [23] compare the four dictionaries (AFFIN, VERDA, Senti-Strength, MPQA) to identify which has a higher or lower potential for the analysis of sentiments in Software Engineering text. Results show that AFFIN performance is better for analyzing SE text whereas MPQA performs poorly due to informal words.

The first paper [24] in which BERT Base is trained on 55000 manually annotated sentences of SO posts. The proposed model is compared with the authors’ previous work, i.e., RNN4SentiSE and SentiMoji. The single sentence-based BERT model achieved 0.87 F-measure. The authors did not compare the proposed model with the domain-specific sentiment analysis tools.

Wu et al. [25] stated that most sentiment analysis tools are trained on movies and product review datasets; therefore, these tools produce negative results when applied to the SE domain. To overcome this limitation, authors fine-tune the BERT Base model (BERT-FT) on GitHub and SO datasets. A comparison of BERT-FT is made with two existing SA tools. The performance of BERT-FT is better than SentiCR and Senti4SD tools. BERT-FT is trained independently on different datasets for comparison purposes. It means that the different BERT models are fine-tuned on six datasets to produce the results.

Batra et al. [26], reported that there is much room to improve existing SE-based sentiment analysis tools because of low F1-score. The authors trained three different models of BERT named BERT-Base, RoBERTa, and ALBERT on GitHub, Jira, and SO datasets and proposed a ranking-based ensemble of BERT models. In addition to this, Barta et al. also used Distil BERT which is a compressed variant of the BERT model. The proposed model achieves 6 to 12% improvements on all datasets. This research’s limitations are that not a single model produces the best results on all the datasets, and the proposed models are not compared with the existing SE domain-specific sentiment analysis tools. The overall F1-score of the authors’ best model is 0.85.

In a recent research, Uddin et al. [27] studied the various SE-specific sentiment analysis tools and observed that no single tool gives satisfactory results. Therefore, the authors proposed the ensemble-based sentiment analysis tool, an ensemble of five existing sentiment analysis tools, and RoBERTa. Prediction of existing sentiment analysis tools and text is input to the RoBERTa. The F1-score of a score of the developed tool is 0.805, whereas the F1-score of the standalone RoBERTa is 0.801.

Mula et al. [28] experiment with various word embedding, features selection, class balance and deep learning classifiers to compute the sentiment of SE text. The purpose of this effort is to propose the best set for sentiment analysis. The authors proposed that the SMOTE sampling technique, ANOVA features selection, and deep neural network with one dropout and two hidden layers improve sentiment analysis tasks.

Published labeled datasets are the main source to train the various classifiers; therefore, the accuracy of the dataset is very important for the sentiment analysis task. Herrmann et al [29] recently surveyed and analyzed the sentiment labels of data from 94 participants and given labels of the dataset. The results of the survey show that 62.5% labels of the dataset coincide with participants and none of the participants agree with the established labels.

Existing sentiment analysis tools work on text data. Developers often communicate in meetings. Therefore, the sentiment of verbal communication is also important. SEnti-Analyzer [30] tools is developed that works well on text and verbal data. The accuracy of this tool is 88 to 90%.

In recent work, Wang et al. [31] proposed a transformer-based multimodal framework named Prototype Augmented Multimodal Teacher Student Network (PAMD) for sentiment analysis. This framework is effective in computing the sentiments when less data is available to train the classifier and there a missing modalities. The model is trained on three different datasets. Two datasets are not related to software engineering and the third dataset is the JavaLib dataset. The model was tested with different missing rates and achieved a maximum accuracy and F1-Score of 0.829 and 0.579 respectively. The results on the JavaLib dataset are not reported by the authors.

In another recently published article, Mark and Andy [32] extracted the software testing-related posts from Stack Overflow and divided these posts into positive and negative posts by using the already developed two sentiment analysis tools (RoBERTa and SentiCR). The authors used grounded theory to further divide the posts into different categories to analyze the emotions of software engineering about software testing. The results show that insecurity, despair, and aspiration impact the software engineering attitude in testing-related posts and they are concerned about the complexity of projects in which testing is applied.

Sing et al. [33], selected four applications from Bugzila and collected bug reports related to these applications, and trained various machine learning classifiers to develop the sentiment analysis tool that prioritizes the bug based on the sentiments of users. The results show that machine learning classifiers save the software engineer time in setting the bug priority level.

The article gives a comprehensive analysis of the technological developments driving Industry 5.0, as well as outlining significant research topics that require additional investigation. By identifying upcoming research prospects such as big data processing, artificial intelligence, drones, cybersecurity, robots, additive manufacturing, IoT, virtual reality, and blockchain, the authors shed light on crucial areas of societal innovation. Furthermore, the article discusses the issues of Industry 5.0, such as the need for a qualified labor, cybersecurity measures, and impediments to technology adoption. This thorough examination of both potential and difficulties provides significant insights for scholars and practitioners alike, directing future efforts to handle the changing landscape of Industry 5.0 [34].

In the existing work, BERT is also being used by researchers for SE domain-specific sentiment analysis, but these studies have various limitations. Like model is trained on one dataset, and comparison with the existing state of the tools is not made [24, 26]. In addition to these limitations, the BERT model is trained on different datasets to show the high accuracy of the model [25]. Furthermore, the existing BERT model works well on trained datasets and cannot be used on general-purpose software engineering resources. From the existing research, it is concluded that sentiment analysis for the software domain is not mature enough, and more attention should be given. Therefore, we decided to use the state-of-the-art BERT model [35] and fine-tune them to compute the sentiment of SE text because BERT has proved its strengths in many NLP tasks, and existing implementations of BERT for SA has various limitations. We also introduced the idea of using the Fuzzy Ensemble of Bert models to combine the strengths of various fine-tuned Bert models to correctly predict the sentiment for the software engineering domain. The proposed model will help to automatically categorize the feedback of users and reduces the workload of software engineers and let them to focus on the innovative tasks instead of labor intensive tasks.

## 3 Proposed methodology

**Fig 1:**
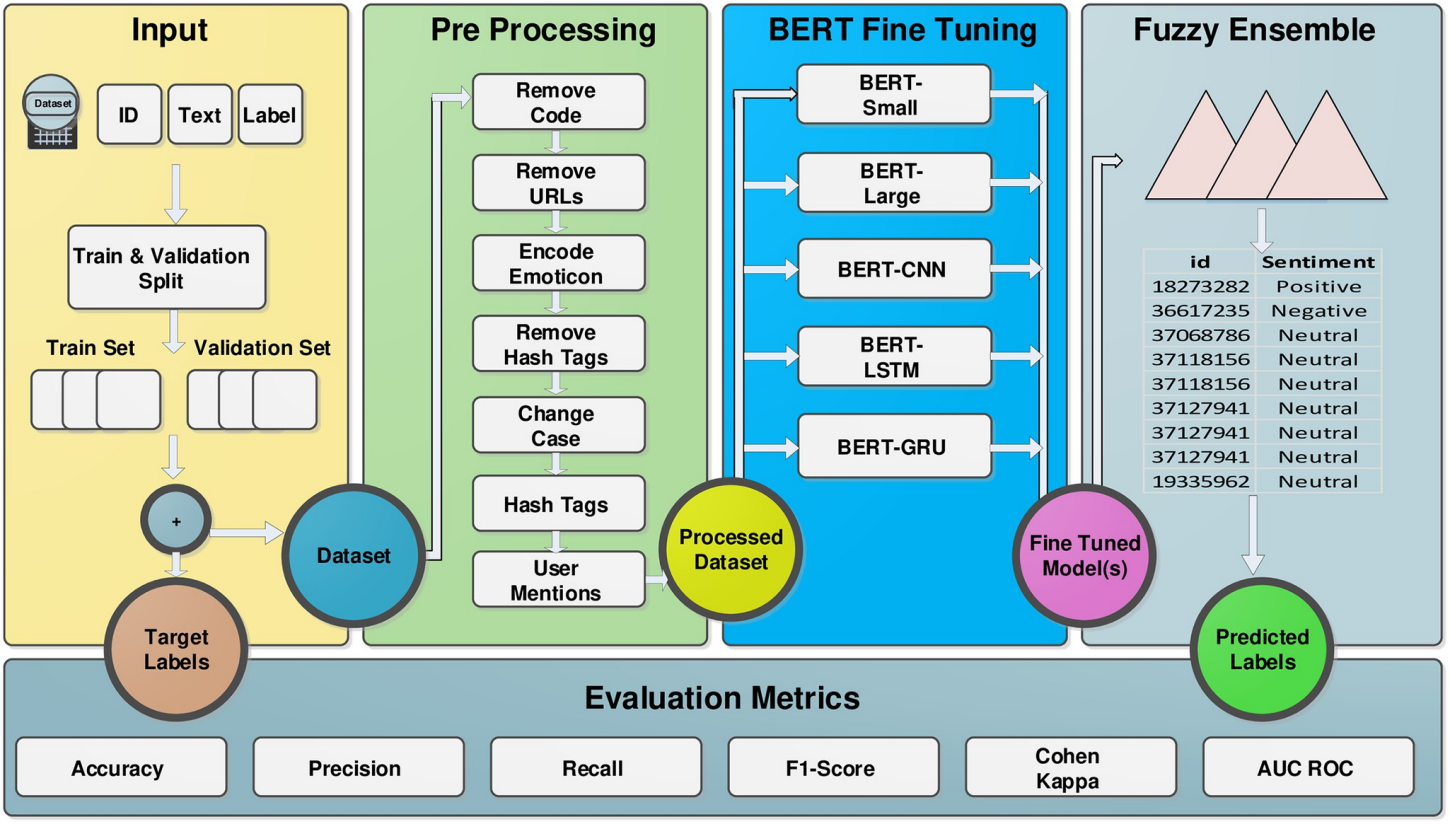
Architecture of the proposed method based on BERT fine tuned models and fuzzy ensemble.

The suggested approach for conducting research is developed to answer the **RQ2** and give us step-by-step procedure to develop domain-specific sentiment analysis tool. The proposed methodology is divided into data pre-processing / cleaning, fine-tuning the BERT models, and developing the fuzzy-based ensemble of fine-tuned models. The layout of the proposed research methodology is presented in Fig 1. Publicly available dataset SentiSE [20] is used to train models in this research therefore, data collection is not part of the proposed methodology.

### 3.1 Sentiment analysis problem definition

Extraction of sentiment from text is an NP-complete problem as expert judgment and decision making is involved in this task. The simplest word counting based pragmatic model of sentiment analysis problem from user post is as defined in this section can be represented by notations given in Table 1.

**Table 1:** Notations to model calculation of sentiments for posts.

Description	Notation
Dataset	*D*_*s*_
Post	*P*
ID	*ID*
Sentiment	*S*
Text	*T*
Words	*W*
Emoji	*EM*

A dataset is a collection of many instances of posts. Here three attributes are associated with post i.e. ID, Sentiment, and Text. Dataset is represented in Eq (1) whereas, an instance of dataset or post is given in Eq (2).

$$\begin{array}{c} D_{s}=\left\{P_{1},P_{2},P_{3},...P_{n}\right\} \end{array}$$ (1)

$$\begin{array}{c} P=\{ID,S,T\} \end{array}$$ (2)

Sentiment or polarity of post may be positive (*S*_*p*_), negative (*S*_*n*_) or neutral *S*_*nu*_ as given in Eq (3).

$$\begin{array}{c} S=\{S_{p},S_{n},S_{nu}\} \end{array}$$ (3)

A post contains positive, negative, and neutral words. If the number of negative words is less than positive words the sentiment of the post is positive. If the number of positive words is less than the number of negative words the sentiment of the post is negative. This can be represented by Eq (4).

$$\begin{array}{c} S=\{\begin{array}{cc} S_{\text{p}}=1 & \text{if}\;N_{w}\leq P_{w};or\\ S_{\text{n}}=-1 & \text{if}\;P_{w}\leq N_{w};or\\ S_{\text{nu}}=0 & \text{if}\;P_{w}=N_{w} \end{array} \end{array}$$ (4)

Text may be a question, answer, or comment that is posted on a community site. It is a collection of words, special characters (*S*_*c*_), numbers (*N*), URLs (*URL*), emojis (*EM*), hashtags (*HT*), user mentions (*UM*), and code (*C*). Text can be represented as Eq (5).

$$\begin{array}{c} T=\{W,S_{c},N,URL,EM,HT,UM,C\} \end{array}$$ (5)

Numbers, special characters alone, URLs, hashtags, user mentions, and code do not add any value for the sentiment analysis task. Therefore, these are removed in the data pre-processing step. After pre-processing our text set can be represented as given in Eq (6):

$$\begin{array}{c} T=\{W,EM\} \end{array}$$ (6)

Words are also classified as positive, negative, and neutral based on their polarity as given in Eq (7).

$$\begin{array}{c} W=\{\begin{array}{cc} P_{\text{w}}=1 & \text{if}\;\text{word}\;\text{is}\;\text{positive};or\\ N_{\text{w}}=-1 & \text{if}\;\text{word}\;\text{is}\;\text{negative};or\\ Nu_{\text{w}}=0 & \text{if}\;\text{word}\;\text{is}\;\text{neutral} \end{array} \end{array}$$ (7)

Emotion or emoji can be positive, negative, or neutral but only positive and negative emojis are encoded in this research because neutral emojis did not contribute to the determination of sentiment. The positive and negative emojis can be represented as Eq (8).

$$\begin{array}{c} EM=\{\begin{array}{cc} P_{\text{EM}}=1 & \text{if}\;\text{emoji}\;\text{is}\;\text{positive};or\\ N_{\text{EM}}=-1 & \text{if}\;\text{emoji}\;\text{is}\;\text{negative}; \end{array} \end{array}$$ (8)

The sentiment of post / text can be calculated from Eq (9). The result of Eq (9) is the sentiment class that can be positive, negative, or neutral. If the post has more positive words or emojis as compared to negative then the result of Eq (9) is positive and vice versa. If the post has no positive and negative words or the number of positive and negative words and emojis is equal then the post will be classified as neutral.

$$\begin{array}{c} S=\sum \left(P_{w}+P_{EM}\right)-\sum \left(N_{w}+N_{EM}\right) \end{array}$$ (9)

### 3.2 Data pre-processing

The publicly available dataset SentiSE [20] is used for training and testing the models. The data is in CSV format and two separate CSV files for training and test data can be downloaded. Each CSV file consists of three columns that are ID, Sentiment, and Text. ID column represents the post ID, the Sentiment column is the sentiment class of the post that may be positive, negative, or neutral and the Text column is the text of the post. The dataset is freely available from SentiSE [20]. As this data is collected from social media it is a noisy dataset because of the casual nature of people. It contains many emojis, user mentions, code, URLs, etc. One has to pre-process data to remove noise from it and convert it to a reduced and normalized form. The accuracy of machine learning classifiers is also dependent on the pre-processing [36]. Therefore, extensive pre-processing setups as given below are performed on the SentiSE and validation dataset [10, 22, 37, 38].

- **Data Cleaning**: Data is converted into lower case, replace two or more dots (.) with space. Strip spaces and quotes from the end of posts and replaces extra spaces with a single space.
- **URL**: Users often share URLs in posts as URLs are not required for sentiment analysis therefore, the URLs are searched with a regular expression (www.[\S]+)|(https?://[\S]+) and replaced with word URL. This expression means any string *S* that is starting with www or https is a URL.
- **User Mention**: Users often mention other users in their feedback. User mentions are usually made by using @handle. Similar to URLs user mentions are also not useful for sentiment analysis therefore, regular expression (@[\S]+) is used to replace user mentions with the word USER_MENTION.
- **Emoticon**: Emoticons are used to express emotions. These emotions are valuable for sentiment analysis. Therefore, a list of emoticons is created and matched in posts. In this step, the positive emoticon is replaced with EMO_POS and negative emoticon with EMO_NEG.
- **Code**: Code also does not contribute to sentiment detection therefore, code is removed from the posts.
- **Hashtag**: Hashtags are not popular in SE community sites but some users may enter hashtags to make the topic trending therefore, the hash (#) symbol is removed from the hashtag by using regular expression #(\S+).
- **Individual Words**: After applying the above pre-processing steps the individual words are processed. If the word starts with the alphabet it is a valid word otherwise it is removed. Punctuation [’”?!,.():;] are also removed from the words. Converted two or more character repetitions to two characters. For example “sooooo” is converted to “soo”.

### 3.3 Fine tuned BERT models for sentiment analysis

Bidirectional Encoder Representations from Transformers (BERT) [39], is a method for pre-training language models. Various BERT models are available freely. These pre-trained models can be used to extract language features from data or can also be fine-tuned for various tasks like classification, entity recognition, question answering, etc. [40] In this paper, BERT is used to train domain-specific sentiment classifiers. This is done by using the two BERT pre-trained models i.e. BERT-Base-Uncased and BERT-Large-Uncased. Fine-tuning is performed by adding an untrained layer of the neuron at the end of the pre-trained model and then retraining the model for the classification task. There are various advantages of fine-tuning BERT. Fine turning is quicker and requires fewer GPU hours. Fine-tuning BERT requires less data as a model is already trained on a huge dataset. For various classification tasks fine-tuned BERT model predicts better results as compared to the training model from scratch.

**Fig 2:**
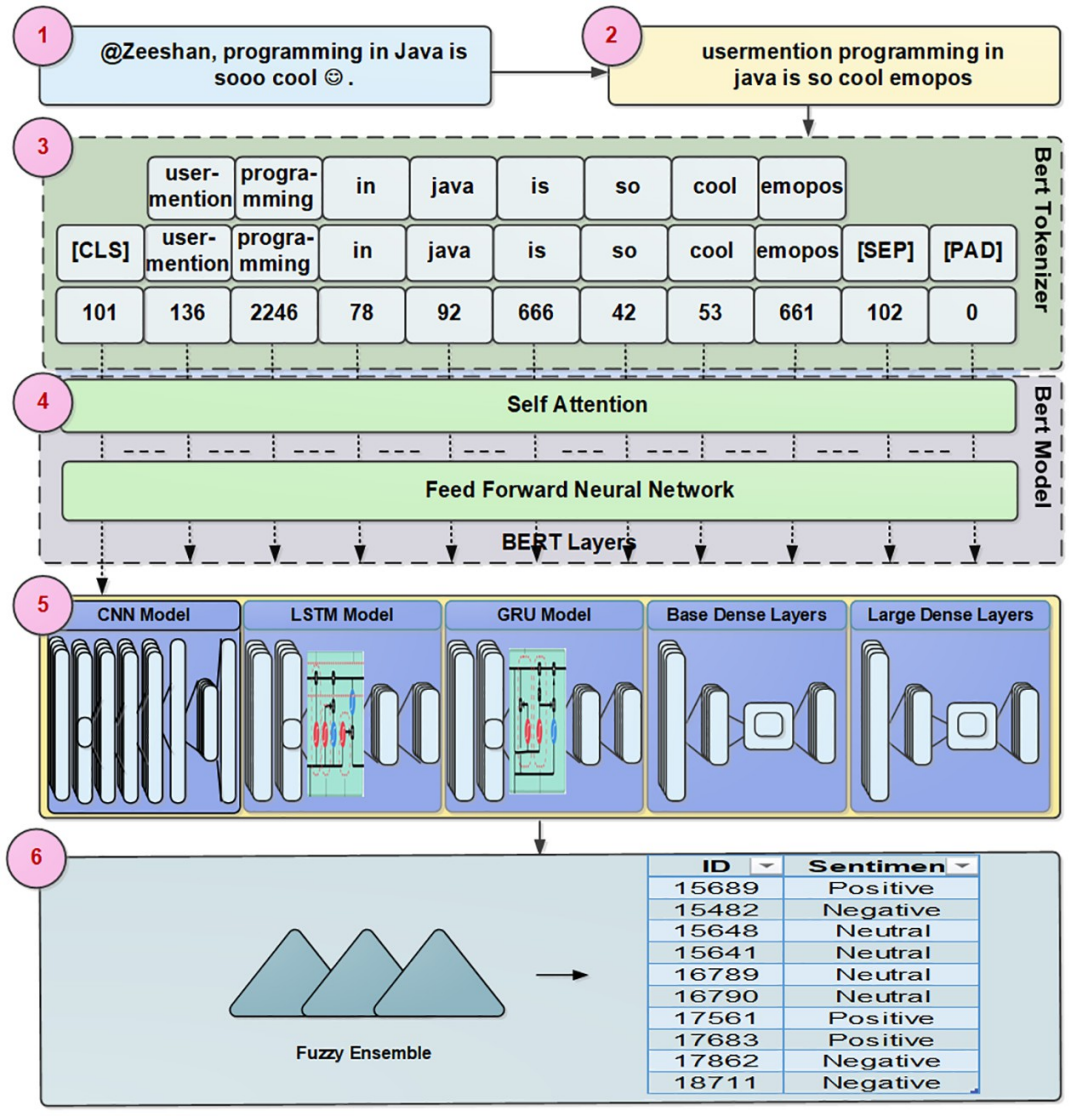
A brief overview of sentiment analysis based on fine tuned BERT models and fuzzy ensemble.

The basic architecture of the fine-tuned BERT model and steps involved for sentiment classification with an example is given in Fig 2. The steps of fine-tuning BERT for sentiment analysis and additional layers involved for this task are labeled 1 to 5 in Fig 2. In the first step user post is shown as input and pre-processing on the post as explained in section 3.2 is performed in step 2. In step 3 post is tokenized by using a BERT tokenizer. In the tokenization process firstly, each word is tokenized, special tokens i.e. CLS, SEP, and PAD are added and finally, an ID is assigned to each token. In step 4 encoding on tokens is performed. There are 12 transformer encoder layers in the BERT-Small and 24 transformer encoder layers in the BERT-Large model. In the 5th step, fine-tuning of the model is performed by inputting the domain-specific dataset to the pre-trained BERT model, adding untrained layers of neurons, and re-training the model to learn the sentiment classification. Different experiments were performed in detail as given in Section 4 in this step by adding layers of Deep Neural Network, LSTM, GRU, and CNN for the creation of a classification model.

### 3.4 Fuzzy ensemble of Bert models

An ensemble based on the Mamdani Type-1 Fuzzy logic mechanism is created. Mamdani’s Type-1 Fuzzy Logic is a mathematical framework for dealing with uncertainty and imprecision in decision-making processes. Fuzzy logic extends crisp logic, which is based on binary true/false values, to handle degrees of truth represented by linguistic variables. In Mamdani’s Type-1 Fuzzy Logic, linguistic variables are used to express subjective or qualitative information. In this article, fuzzy logic combines the results of various fine-tuned Bert Models to compute the sentiment of the dataset. The advantage of this ensemble is that it combines the strength of each algorithm through fuzzy rules. The architecture of the proposed fuzzy ensemble is based on the five inputs and one output. Input to the fuzzy ensemble is the output of the fine-tuned BERT model. As in this research, five different variants of the BERT model are used, and the output of each BERT model is given as input to the fuzzy ensemble model. Each input of the fuzzy ensemble is further divided into three membership functions. The Trapezoid membership function represents the positive and neutral sentiments as the Gaussian membership function is used for neutral. Similarly, three Gaussian membership functions are used for the smooth representation of the fuzzy output. The membership function defines the degree to which an element belongs to a particular class. The output is the sentiment class of the instance of data. Membership functions of Bert models and their parameters are given in Table 2. The fuzzy model predicts the outcome by using fuzzy rules. The fuzzy rules are conditional statements that are evaluated to reach a crisp value. In the proposed fuzzy ensemble, maximum possible fuzzy rules were created. The values of fuzzy membership function parameters and rules firing strength are fine-tuned by utilizing Genetic Algorithm (GA). Tuning the fuzzy membership function parameters and rule-firing strength is a critical step in enhancing the effectiveness, adaptability, and interpretability of fuzzy logic systems, ultimately leading to improved performance for predicting values. Five generations of the population were created by using a genetic algorithm and parameters with minimum root mean square error were selected for predicting sentiment in the proposed fuzzy ensemble.

**Table 2:** Fuzzy membership function parameter values.

Name	Bert-Models	Output
Range	[0 2]	[0 2]
Negative	Trapezoid [0 0 0.8 1]	Gaussian [0.09579 0]
Neutral	Gaussian [0.05 1]	Gaussian [0.105 0.9935]
Positive	Trapezoid [1 1.2 2 2]	Gaussian [0.105 2]

### 3.5 Evaluation metrics

To check the prediction models, accuracy, precision, recall, F1-Score, Cohen Kappa, and AUC ROC of each model are calculated. The best value of these metrics is 1. This means the model is 100% accurate.

## 4 Experiments and analysis

Five variants of Bert are fine-tuned in this research. In the first two variants, pre-trained Bert models (Bert-Base and Bert-Large) are modified, and added layer of neurons for sentiment analysis. In the next two experiments, two variants of Recurrent Neural Network (RNN) are used and added layers of Longterm Short Term Memory Network (LSTM) and Gated Recurrent Units (GRU) are over the Bert-Base model for the classification of sentiments. In the last experiment, Convolution Neural Network (CNN) and Bert are used for sentiment classification.

10% of training data is used for validation to avoid over-fitting i.e. 8508 posts for training and 945 posts for validation are used. 4052 posts are used for testing. For each model, accuracy, precision, recall, F1-score, Cohen Kappa, and ROC AUC are calculated. The details of experiments for best Bert model selection and results on validation and test dataset are given below:

### 4.1 Experiment 1: Fine tuning CQA-BERT

In the first two experiments, the Hugging Face library [41], which is the PyTorch interface for working with BERT is utilized. In addition to pre-trained transformer models, this library also includes several pre-built task-specific modifications of these models. Bert for the Sequence Classification model is fine-tuned for domain-specific sentiment analysis. In this model, a single layer of neurons is added at the top of BERT for classification. When domain-specific data is fed the entire BERT and classification layer is trained for a specific task.

In the experiment, the data is tokenized with a BERT tokenizer, added SEP & CLS tokens at the start and end of sentences, and pad the tokens by adding special [PAD] tokens to a fixed length. Experiments were performed with different sentence lengths i.e. 64, 128, 256, and 512. An Attention mask is also used to differentiate the real tokens from padded tokens. Padded tokens differentiated in attention masks are not used by the BERT self-attention mechanism for the interpretation of sentences. Training data is divided into 90% training set and 10% validation set. The training hyperparameters of CQA-BERT are given in Table 3. The values of these parameters were set in the Python code of the model. In order to optimize the performance of various BERT models, a meticulous process of hyperparameter tuning was undertaken. Hyperparameters play a crucial role in the configuration of machine learning models, influencing their learning capacity and generalization ability. In this study, we systematically explored and adjusted hyperparameters by conducting repeated experiments and manipulating different values. The primary objective was to identify the combination of hyperparameter values that maximized the accuracy of each BERT model on a subset of our dataset. This subset was carefully chosen to serve as a representative sample for tuning, allowing for more efficient exploration of hyperparameter space without the computational burden of using the entire dataset. Through an iterative process of experimentation and fine-tuning, we systematically changed hyperparameter values, such as learning rates, batch sizes, and dropout rates, among others. The performance of each model was measured based on accuracy, with multiple iterations performed to ensure robustness and reliability of results. Ultimately, the chosen hyperparameter values represent the configuration that yielded the highest accuracy for each BERT model.

**Table 3:** CQA-BERT control parameters.

Parameter	Value
Model	BERT_Base_Uncased, BERT_Large_Uncased
Batch Size	32
Max_Length	64, 128, 256, 512
Learning Rate	2e-5
Epochs	4
Epsilon parameter	1e-8
Optimizer	AdamW

Google Colaboratory [42] is used for implementation in all experiments with BERT. Authors fine-tune both BERT_Base_Uncased and BERT_Large_Uncased models for domain-specific sentiment analysis. Models were trained on different sentence lengths i.e. 64, 128, 256, and 512. The model’s accuracy across different sentence lengths was calculated for example accuracy of the BERT-Base model on a test dataset with a sentence length of 64 is 0.825, and the accuracy with 256 sentence length is 0.846 and the accuracy with 512 sentence length is 0.846. From the results of different sentence lengths, it is observed that increasing sentence length does not considerably improve the accuracy of the model therefore, 256 sentence length is used in the rest of the experiments. The accuracy of both fine-tuned models on the validation dataset is 0.850 whereas the test data accuracy of the Bert-Base model is 0.846 with a sentence length of 256 is higher than the Bert-Large model. Precision, recall, and F1-Score metrics consistently reflected the strong performance of both models. The Cohen’s Kappa coefficient, measuring inter-rater agreement, was 0.722 for Bert-Base and 0.723 for Bert-Large, indicating substantial agreement. Additionally, the area under the receiver operating characteristic curve (AUC) values further affirmed the models’ discriminative power, with Bert-Base achieving an AUC of 0.861 and Bert-Large close behind at 0.867. These comprehensive results underscore the robustness of both Bert-Base and Bert-Large, with Bert-Base exhibiting a slight advantage in accuracy.

### 4.2 Experiment 2: Fine tuning BERT-RNN

Two variants of the Bert-Recurrent Neural Network (Bert-RNN) i.e. Bert Long Term Short Term Memory Network (Bert-LSTM) and Bert Gated Recurrent Unit (Bert-GRU) are implemented in this experiment. These models treat sentiment analysis as a multi-classification problem and predict the sentiment of given posts. Google Colab [42] platform is used for implementation in Python and Bert, Tensorflow, and Keras libraries are utilized for implementation of Bert-RNN. First of all, Bert is fine-tuned on training data as described in article [39] and google official tutorial [35]. After fine-tuning the Bert, output pooled is extracted which is the representation vector of size 768 for every chunk. The representation vector is input to RNN (LSTM and GRU) after padding and masking it. The model is trained with Kera’s callback (ReduceLROnPlateau) that reduces the learning rate if validation accuracy does not improve. A summary of the RNN model (LSTM and GRU) is given in Table 4. The values of these parameters were fixed in the Python code of the model.

**Table 4:** RNN model control parameters.

Layer (type)	Output Shape	Param #
text (Input Layer)	(None, None, 768)	0
masking_3 (Masking)	(None, None, 768)	0
lstm_3 (LSTM/GRU)	(None, 100)	347600
dense_5 (Dense)	(None, 30)	3030
dense_6 (Dense)	(None, 3)	93

The results of Bert-LSTM and Bert-GRU models are presented in Table 5. Notably, Bert-LSTM exhibits a slight advantage over Bert-GRU, with an accuracy of 0.861 on the validation dataset and 0.848 on the test dataset. This nuanced superiority suggests that the Long Short-Term Memory (LSTM) architecture, when combined with Bert embeddings, contributes to a more effective model than the Gated Recurrent Unit (GRU).

**Table 5:** Results of different BERT variants on the test dataset.

Algorithm	Accuracy	Precision	Recall	F1	C Kappa	AUC
CQA-Bert-Base	0.846	0.823	0.818	0.820	0.722	0.861
CQA-Bert-Large	0.845	0.816	0.827	0.821	0.723	0.867
Bert-LSTM	0.848	0.844	0.792	0.812	0.715	0.843
Bert-GRU	0.845	0.840	0.785	0.808	0.707	0.838
Bert-CNN	0.792	0.747	0.773	0.789	0.686	0.858
Fuzzy Ensemble	0.851	0.836	0.811	0.822	0.728	0.857

A notable comparison was made by implementing LSTM with both Bert and Glove embeddings to assess their respective efficiencies. Strikingly, the results reveal a 12% improvement in accuracy when using Bert embeddings, underlining the superior performance of Bert-LSTM over its Glove-based variant. This disparity emphasizes the potency of leveraging Bert embeddings to enhance the capabilities of LSTM models. The observed differences in accuracy underscore the importance of embedding choices and their impact on the overall effectiveness of the models. Further investigations into the specific contexts and tasks where Bert-LSTM excels could provide valuable insights into the strengths and limitations of this architecture.

### 4.3 Experiment 3: Fine tuning BERT-CNN

Bert Convolutional Neural Network (Bert-CNN) is also implemented using Google Colab [42] and TensorFlow. Post text is first tokenized with Bert Tokenizer so that Bert text embedding can be used. The Bert embedding layer is created by importing the Bert model from the hub.KerasLayer. The classification model consists of four convolution neural network layers. Each layer is initialized with a filter size of 2, 3, 4, and 5 respectively. Global max pooling is applied to the output of each layer of CNN. Then the output of each layer of CNN is concatenated and passed to a dense neural network. The prediction is made with a second dense neural network and it predicts sentiment into three classes. The model trained with Kera’s callback (ReduceLROnPlateau) reduces the learning rate if validation accuracy does not improve. The hyperparameters of the Bert-CNN model are listed in Table 6 and the values of these parameters were fixed in the Python code of the model.

**Table 6:** The control parameters values of BERT-CNN.

Parameter	Value
Model	BERT_Base_Uncased
Batch Size	32
EMB_DIM	200
VOCAB_LENGTH	len(tokenizer.vocab)
CNN_FILTERS	300, 150, 100, 75
DNN_UNITS	256
Output Classes	3
Dropout	0.2
Epochs	10

The development of the Bert-CNN model involved a systematic exploration of various configurations, including different numbers of Convolutional Neural Network (CNN) layers, filters, kernel sizes, and dense units. After a thorough evaluation, the optimal set of parameters is outlined in Table 6. The results of the Bert-CNN model are listed in Table 5, revealing valuable insights into its effectiveness.

The Bert-CNN model achieved an accuracy of 0.811 and 0.792 on validation and test dataset respectively, showcasing its competency in understanding and classifying textual information. Notably, the choice of CNN architecture parameters significantly influences the model’s performance. The results underscore the importance of careful hyperparameter tuning to harness the full potential of the Bert-CNN model.

A critical analysis of the results suggests that while the accuracy on the test set is slightly lower than that on the validation set, the Bert-CNN model exhibits robust performance. It’s essential to consider potential overfitting or generalization issues and further investigate strategies to enhance model generalization to unseen data. Additionally, insights gained from parameter tuning and performance evaluation contribute to a deeper understanding of the Bert-CNN model’s strengths and areas for potential improvement. Future work could explore additional architectural variations and fine-tuning strategies to continue optimizing the Bert-CNN model for specific tasks and datasets.

### 4.4 Fuzzy ensemble of Bert models

The results of the five fine-tuned Bert models were ensemble using the fuzzy ensemble model. The detail of the fuzzy ensemble model is given in section 3.4. The output of each Bert Model is given as input to the fuzzy ensemble model. The output of the fuzzy ensemble model is the sentiment class. The fuzzy ensemble model results are given in Table 5. The accuracy of the fuzzy ensemble model on the test dataset is 0.851 that is higher than all of the individual Bert models.

This section answers the **RQ3** and shows to what extent the proposed fuzzy ensemble of BERT models overcomes the limitations of the existing sentiment analysis tools for the software domain. The existing tools are trained on a specific software engineering dataset and report the results on the same dataset. The performance of the existing tool on the new dataset is very low. Therefore, the existing tools cannot be used as a general-purpose resource to compute the sentiment of software engineering datasets. The same is also highlighted by Novielli et al. [12]. To address this issue, we trained all the models on the SentiSE dataset and reported the predicted score on different already published datasets. The performance of the proposed fuzzy ensemble is not only comparable with the existing tools but it is better than the existing tools on Stack Overflow, Jira, and CodeReview datasets.

The results indicate that both individual BERT models and the proposed fuzzy ensemble model demonstrate notable performance in predicting neutral and positive sentiment classes. This achievement addresses a common limitation observed in existing models that often struggle with accurate predictions for neutral sentiment. Specifically, the accuracy for predicting the neutral class ranges impressively from 80% to 90%, showing the robustness of the models in capturing the neutral sentiments.

Similarly, the accuracy of the models in predicting positive sentiments is commendable, with rates ranging from 83% to 85%. This suggests a consistent ability to identify and classify positive sentiment expressions within the dataset. However, it’s noteworthy that the accuracy of the fuzzy ensemble model in predicting negative sentiments is comparatively lower at 68%. This lower performance in the negative class prediction is attributed to the challenges posed by GRU, CNN, and LSTM-based variants of BERT models in handling negative sentiment.

**Fig 3:**
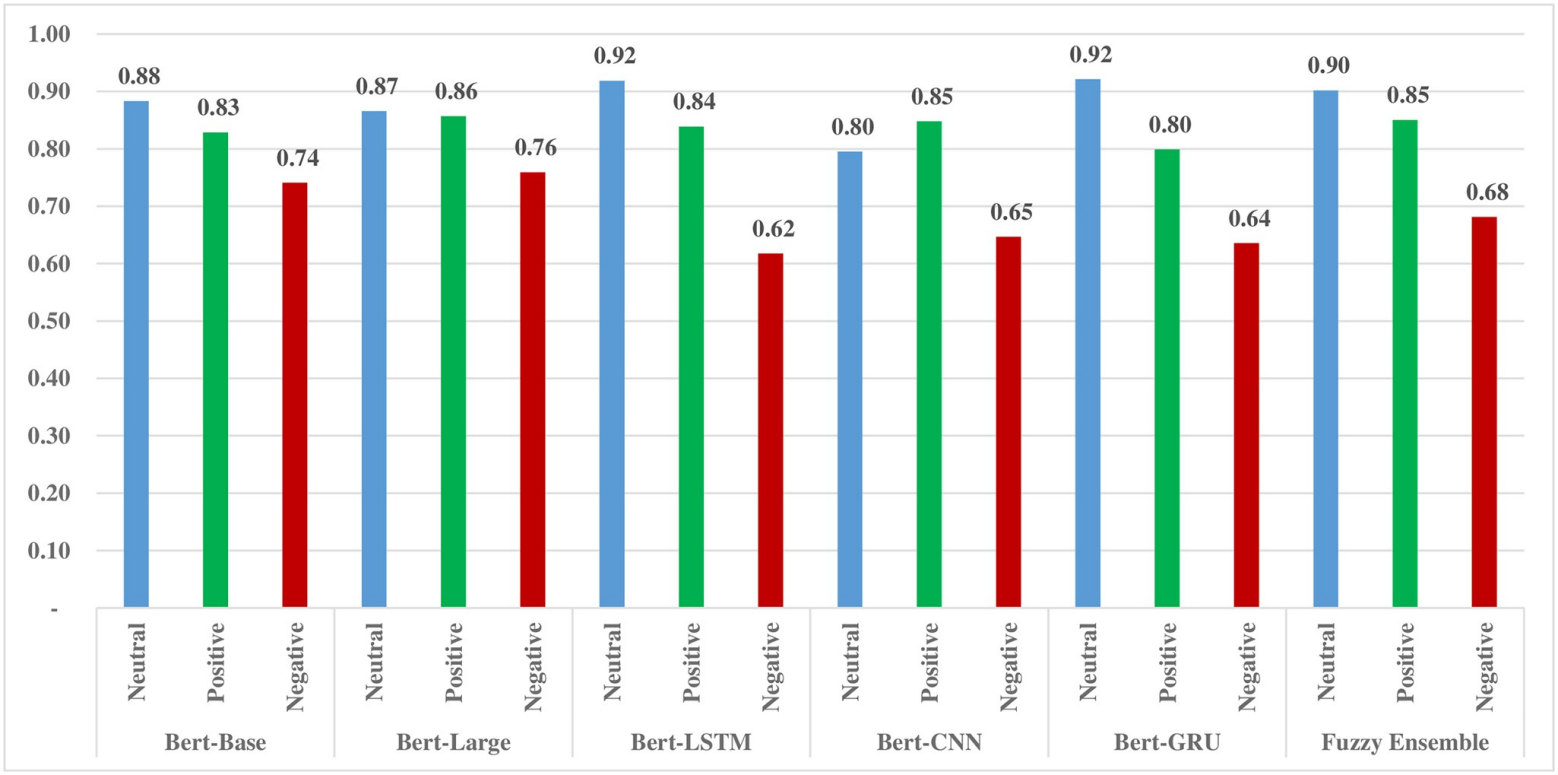
Comparative analysis of BERT models and fuzzy ensemble to predict sentiment classes on test dataset.

The incorporation of a fuzzy ensemble mechanism provides a holistic approach to sentiment analysis, effectively leveraging the strengths of multiple BERT models. The comparison of accuracy rates for positive, negative, and neutral classes is graphically presented in Fig 3, offering a visual summary of the models’ performance across different sentiment categories. These findings collectively underscore the efficacy of the ensemble approach in achieving robust sentiment prediction, particularly in handling the often challenging neutral sentiment class.

### 4.5 Statistical analysis of results

**Fig 4:**
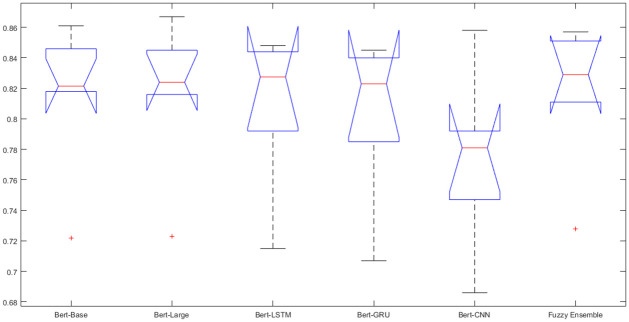
Analysis of results using one way ANOVA.

In addition to absolute analysis of results, Statistical analysis is also performed by using Analysis of Variance (ANOVA) and Effect Size. ANOVA is applied to assess the statistically significant differences in the mean of the fine-tuned Bert-Models and Fuzzy Ensemble model. In this case, the value of p is 0.684. Since the value of *p* > 0.05, this rejects the null hypothesis and implies that there is insufficient evidence to conclude that there are significant differences in the evaluated metrics among the six models. The box plot of ANOVA is given in Fig 4. From the box plots, it is observed that the mean of five out of the six models i.e. Bert-Base, Bert-Large, Bert-LSTM, Bert-GRU, and Fuzzy Ensemble is very close. However, the mean of the Bert-CNN model is lower than the other models. This implies that there is a further need to perform statistical analysis by using other techniques. The most suitable technique in this particular case is Effect Size analysis.

**Fig 5:**
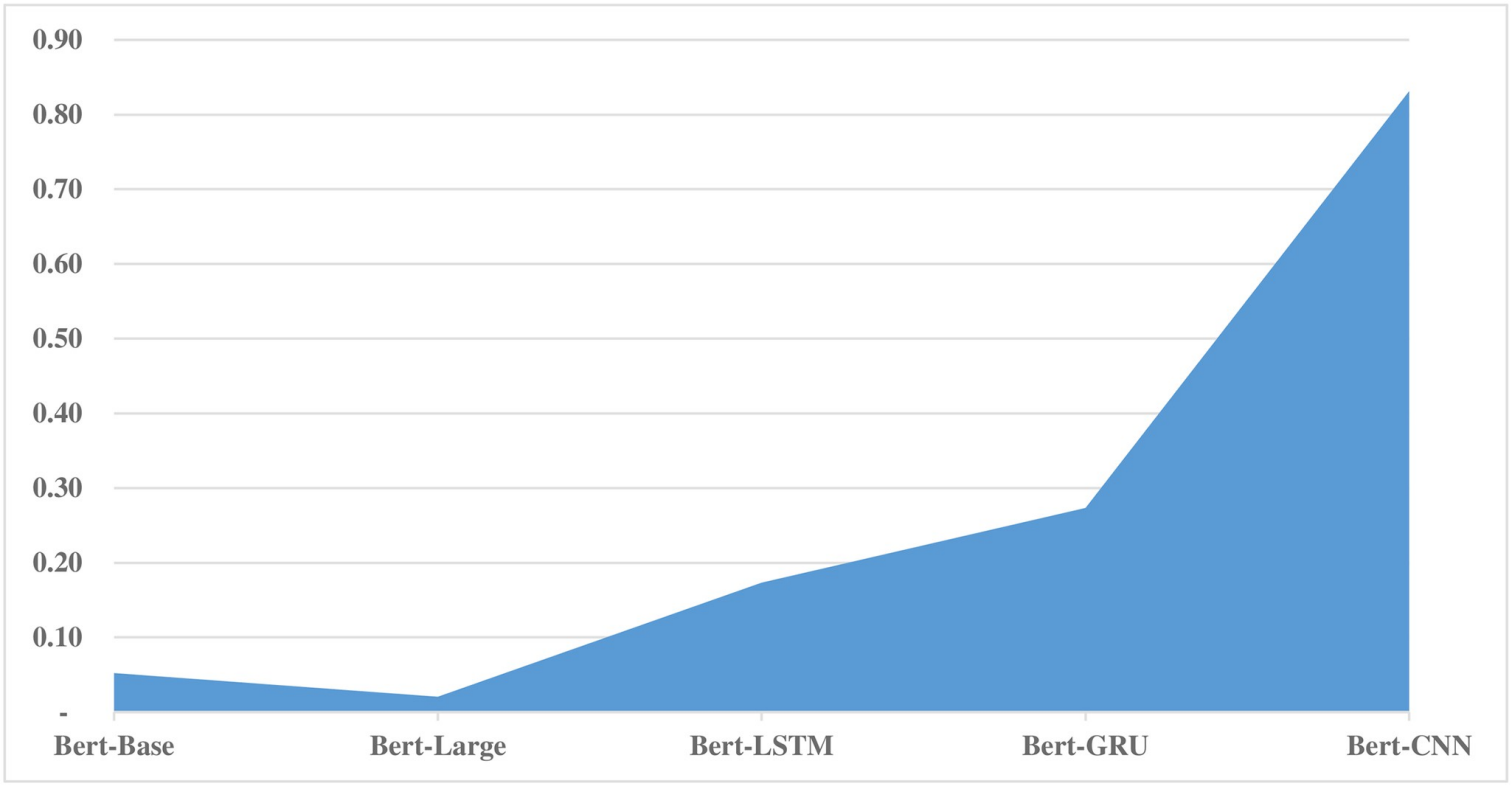
Effect size analysis of fine tuned Bert models with fuzzy ensemble.

Effect size is used to compare the two or more models. Effect sizes provide a measure of practical significance, helping to understand the extent of the observed differences beyond statistical significance. In this particular case, the Fuzzy Ensemble model is compared with all the fine-tuned Bert models. The value of effect size tells the difference among models. An effect size of 0.052, particularly when referring to the comparison between Bert-Base and Fuzzy-Ensemble, quantifies the magnitude of the difference between these two groups. A small effect size suggests that while there is a statistically significant difference between Bert-Base and Fuzzy-Ensemble, the practical significance of this difference may be limited. The results of the effect size analysis are given in Fig 5. From the results, it can be observed that the Bert-Base and Bert-Large models are closest to the Fuzzy Ensemble, whereas, Bert-LSTM, Bert-GRU, and Bert-CNN are quite different.

## 5 Discussion

In this section, the proposed model is evaluated on the various published software engineering datasets to judge the effectiveness of the fine-tuned language models on the diverse datasets. The comparison of the proposed model with existing cutting-edge sentiment analysis tools is also presented in this section. In addition to this, consequences of fine-tuning the large language models are also discussed in this section. Finally, practical application of the proposed Fuzzy Ensemble framework to reduce the labor intensive work of software engineers is also briefly explained in this section.

### 5.1 Fuzzy ensemble model validation

The Fuzzy Ensemble model is validated with different publicly available sentiment datasets [10, 22, 37, 38] related to software engineering. The data is in CSV format that can be downloaded from given sources. Each CSV file consists of three columns that are ID, Sentiment, and Text. ID column represents the post ID, the Sentiment column is the sentiment class of the post that may be positive, negative, or neutral and the Text column is the text of the post. The dataset is freely available from the above sources. An overview of each dataset is given in Table 7. In Table 7, statistics of each dataset like number of posts, emotions, user mentions, and number of URLs along with the number of positive, negative, and neutral sentiments are given. These validation datasets were also pre-processed before input into the fine-tuned models. The results of fine-tuned model prediction on these datasets are evaluated using evaluation metrics as given in Section 3.5.

**Table 7:** Benchmark dataset used for validation of fuzzy ensemble model.

Dataset	Attribute	Total	Positive	Negative	Neutral
Stack Overflow	Posts	4423	1525	1204	1694
	Emotions	400	288	112	-
	User Mentions	236	-	-	-
	URLs	185	-	-	-
JavaLib	Posts	1500	131	178	1191
	Emotions	3	0	3	-
	User Mentions	9	-	-	-
	URLs	-	-	-	-
Jira	Posts	2565	1103	760	702
	Emotions	132	118	14	-
	User Mentions	49	-	-	-
	URLs	71	-	-	-
CodeReview	Posts	1600	1202	398	-
	Emotions	88	61	27	-
	User Mentions	32	-	-	-
	URLs	73	-	-	-

#### 5.1.1 Fuzzy ensemble validation on Stack Overflow dataset

Stack Overflow is a community question answering site that is dedicated to programming questions. Calefato et al [22], created a sentiment dataset of Stack Overflow that consists of 4423 posts. An overview of the Stack Overflow dataset is given in Table 7.

**Fig 6:**
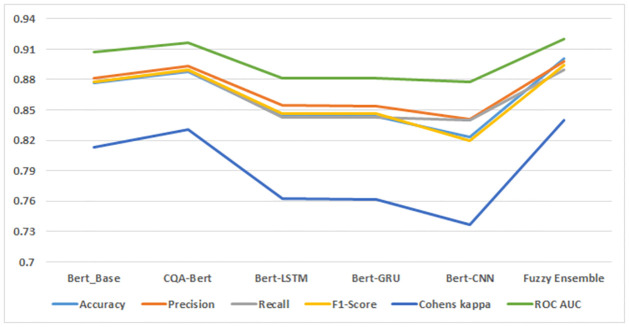
Fine tuned and ensemble model results on Stack Overflow dataset.

The experimental evaluation on the Stack Overflow dataset using fine-tuned BERT models reveals compelling insights into the performance of our proposed Fuzzy Ensemble model, as illustrated in Fig 6. Notably, the Fuzzy Ensemble model achieves an outstanding accuracy of 0.901, surpassing the individual Bert models in classification accuracy. This signifies the efficacy of our ensemble approach in combining the strengths of multiple fine-tuned BERT models.

Furthermore, the recall metric, which measures the model’s ability to correctly identify positive instances, attains an impressive value of 0.898 for our proposed model. This result outperforms all individual Bert models, highlighting the robustness of our ensemble in capturing relevant sentiments effectively. The F1-Score, a harmonized metric of precision and recall, reaches a commendable 0.894, demonstrating the balanced performance of our proposed model compared to the individual Bert models.

Cohen’s Kappa, an inter-rater agreement metric, attains a notable value of 0.840 for our proposed model, surpassing the performance of all individual Bert models. This indicates a high level of agreement between the predictions of our model and the ground truth labels. Finally, the Area Under the Curve (AUC) metric, a measure of the model’s ability to distinguish between positive and negative instances, achieves an excellent value of 0.920, outperforming all individual Bert models.

In summary, the comprehensive analysis of accuracy, recall, F1-Score, Cohen’s Kappa, and AUC collectively supports the conclusion that our proposed Fuzzy Ensemble model consistently outperforms individual Bert models in classifying sentiment within the Stack Overflow dataset. These results affirm the effectiveness of our ensemble approach in enhancing sentiment analysis outcomes.

#### 5.1.2 Fuzzy ensemble validation on JavaLib dataset

JavaLib dataset was created by Lin et al [10]. This dataset is extracted from Stack Overflow and is composed of randomly selected sentences that are extracted from posts related to JavaLib. The dataset is in CSV format. JavaLib dataset was labeled by five researchers into positive, negative, and neutral sentiments. Labeled JavaLib dataset can be downloaded from [20]. This dataset consists of 1500 posts. An overview of the JavaLib dataset is given in Table 7.

**Fig 7:**
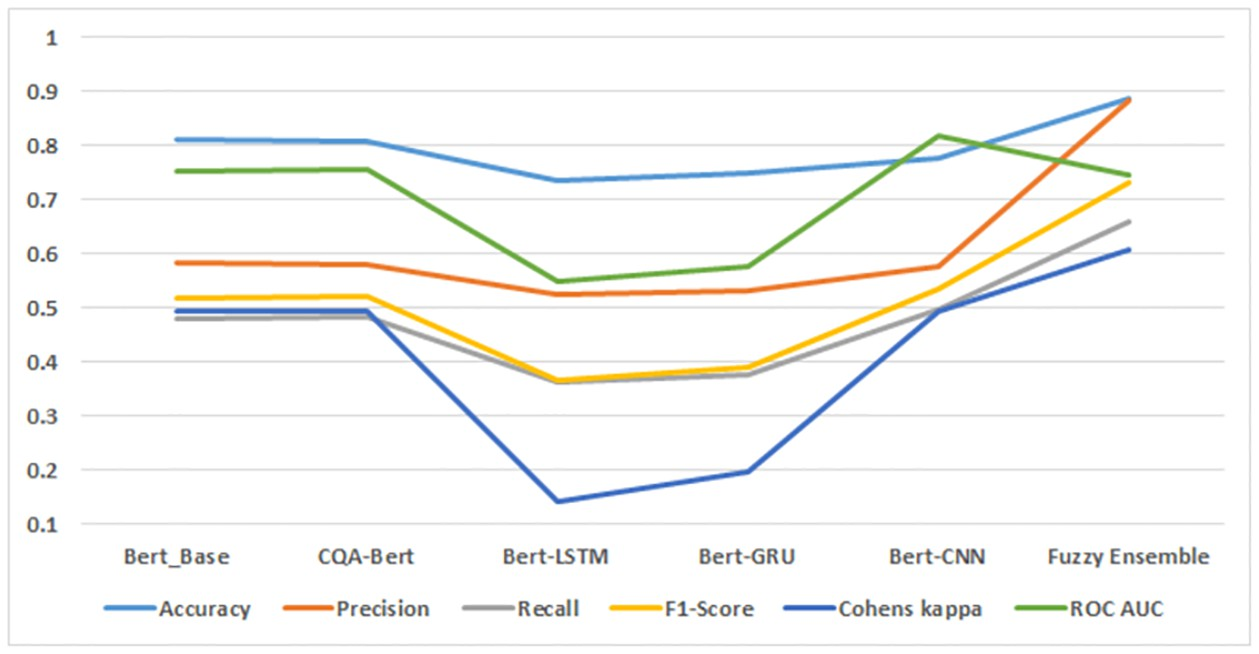
Fine tuned and ensemble model results on JavaLib dataset.

The evaluation of five fine-tuned BERT models and proposed Fuzzy Ensemble model on the JavaLib dataset, as depicted in Fig 7, provides valuable insights into their respective performance metrics. Notably, the Fuzzy Ensemble model emerges as a standout performer, achieving an accuracy of 0.888. This accuracy surpasses all individual classifiers, indicating the effectiveness of our ensemble approach in capturing sentiment nuances within the JavaLib dataset.

Examining precision, the Fuzzy Ensemble model attains a commendable value of 0.883. Precision reflects the model’s capability to accurately detect positive cases among those predicted as positive, emphasizing the reliability of our proposed approach. The recall metric, with a value of 0.659, is higher for our proposed model compared to all other classifiers. While this signifies despite the model’s capacity to catch a significant fraction of positive events, the trade-off between precision and recall is clear and should be considered in the context of specific application requirements.

The F1-Score, a balanced metric of precision and recall, achieves a noteworthy value of 0.733 for our proposed model, outperforming all other classifiers. This underscores the model’s ability to achieve a harmonized trade-off between precision and recall, indicating a balanced classification performance. Cohen’s Kappa, measuring inter-rater agreement, reaches a value of 0.609 for our proposed model, surpassing individual classifiers and signifying a substantial level of agreement between predictions and ground truth labels.

Finally, the Area Under the Curve (AUC) metric, indicative of the model’s discriminatory power, attains a commendable value of 0.747 for our proposed Fuzzy Ensemble model. This result suggests superior performance in distinguishing between positive and negative instances compared to individual classifiers.

In conclusion, the comprehensive analysis of accuracy, precision, recall, F1-Score, Cohen’s Kappa, and AUC collectively supports the assertion that our proposed Fuzzy Ensemble model consistently outperforms individual classifiers in sentiment classification on the JavaLib dataset.

#### 5.1.3 Fuzzy ensemble validation on Jira dataset

Jira is an issue-tracking system, where developer posts their issues and comments. The Jira dataset was created by Ortu et al [37], it consists of issue comments posted by developers about popular open-source software like Apache, Spring, Jboss, and CodeHaus. The Jira dataset can be downloaded from [20]. This dataset is composed of 2565 issue comments. An overview of the Jira dataset is given in Table 7.

**Fig 8:**
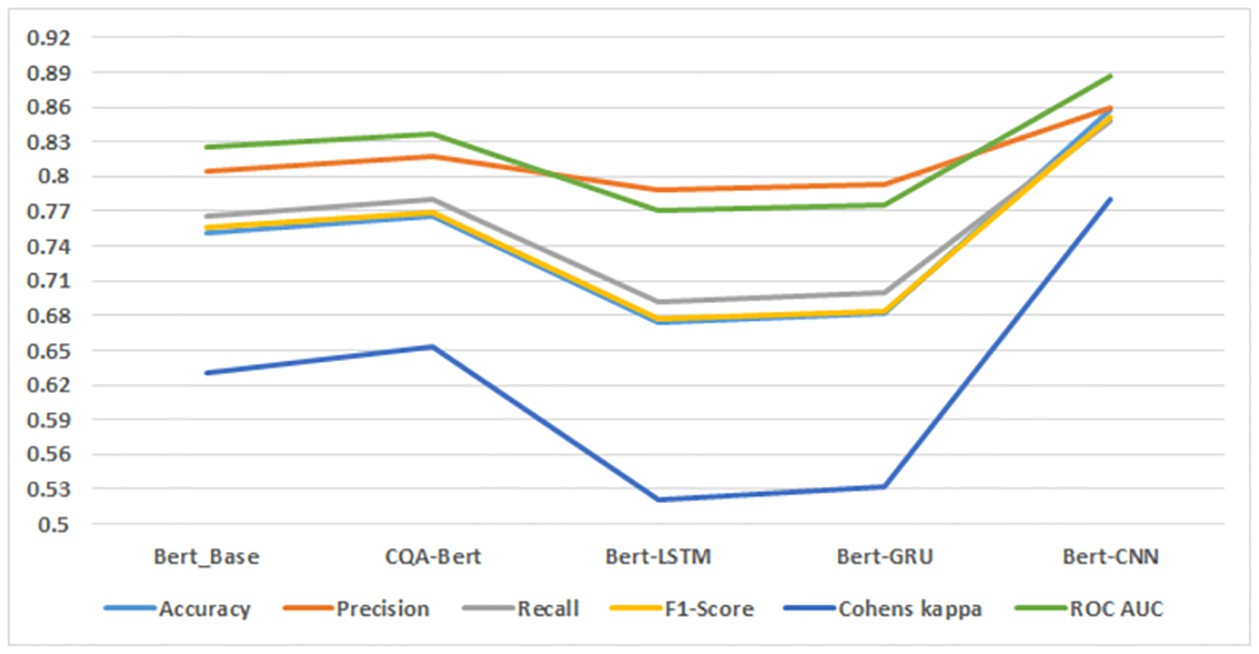
Fine tuned and ensemble model results on Jira dataset.

The classification results of fine-tuned models on the Jira dataset, illustrated in Fig 8, present a nuanced picture of model performance. The accuracy of the Fuzzy Ensemble model on the Jira dataset is noteworthy at 0.759. While this accuracy is higher than the three Bert models, it falls short of the performance achieved by Bert-Large and Bert-CNN. It is essential to recognize that accuracy alone may not capture the full complexity of the model’s performance, and a more in-depth analysis is required.

Interestingly, the Fuzzy Ensemble model exhibits higher precision compared to four out of the five proposed Bert models, indicating its ability to minimize false positives. Precision is a crucial metric, particularly in scenarios where the cost of false positives is high, and these results suggest the Fuzzy Ensemble’s suitability in such contexts.

Examining the F1-Score, a metric that balances precision and recall, the Fuzzy Ensemble model demonstrates superior performance compared to the three proposed Bert models. This underscores the ensemble’s ability to achieve a balanced trade-off between precision and recall, contributing to a more robust overall performance.

Despite the commendable performance of the Fuzzy Ensemble model, it’s important to note that the Bert-CNN model emerges as the most suitable for classifying the Jira dataset. This suggests that the convolutional neural network architecture, as applied to BERT embeddings, offers distinct advantages in capturing the complex patterns present in Jira dataset sentiments.

In summary, while the Fuzzy Ensemble model delivers competitive results on the Jira dataset, the nuanced performance landscape suggests that the choice of the most suitable model depends on specific task requirements. The Bert-CNN model, with its high accuracy, might be preferred for certain scenarios, emphasizing the need for careful consideration of the trade-offs between different performance metrics and the task at hand.

#### 5.1.4 Fuzzy ensemble validation on CodeReview dataset

The CodeReview dataset was created by Ahmed et al [38] for the code repository of twenty open-source projects. The developer’s comments from the code repositories are collected and labeled as positive and negative. There are 1600 comments in the CodeReview dataset. Statics of the CodeReview dataset are given in Table 7.

**Fig 9:**
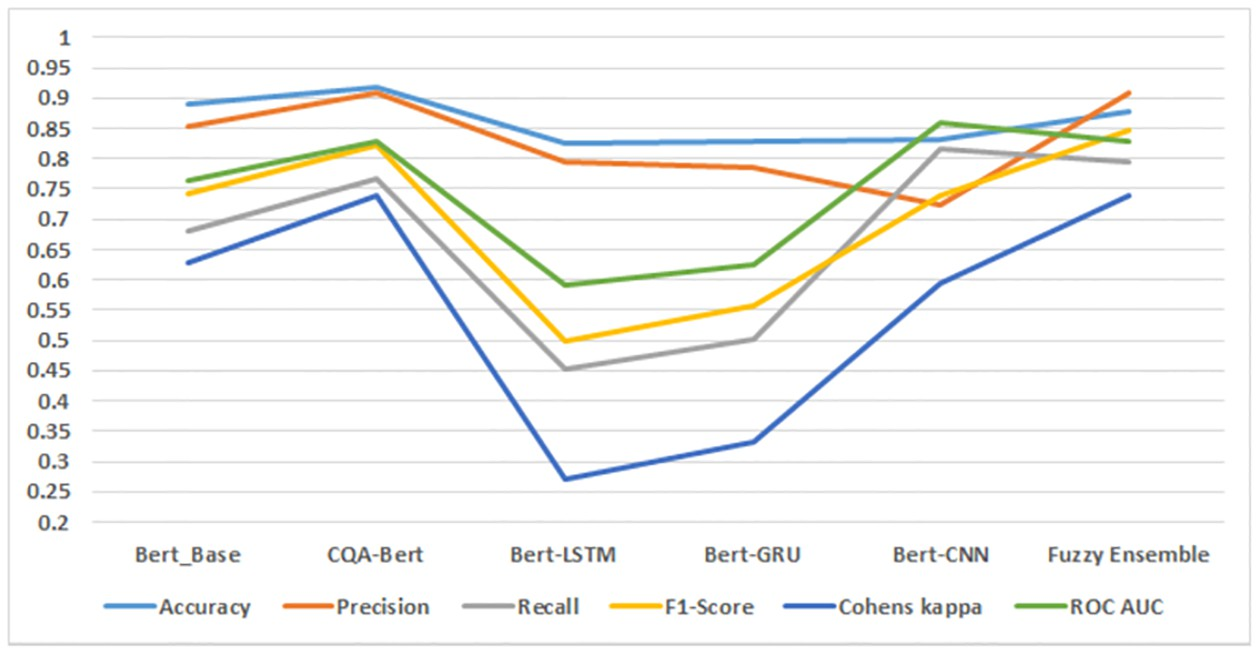
Fine tuned and ensemble model results on CodeReview dataset.

The classification results of fine-tuned models on the CodeReview dataset, as presented in the Fig 9, reveal nuanced insights into their performance. Notably, the Bert-Base model achieves a commendable accuracy of 0.889, demonstrating its ability to correctly classify instances in a binary sentiment setting. However, the precision and recall metrics suggest that while the model is accurate, it may struggle with capturing the full scope of positive instances, as evidenced by the precision of 0.854 and recall of 0.681.

The CQA-Bert model exhibits superior overall performance, with an accuracy of 0.919, demonstrating its effectiveness in binary sentiment classification. The high precision (0.908) and recall (0.767) values indicate a well-balanced ability to correctly identify both positive and negative instances. The model’s F1-Score of 0.822 further underscores its balanced performance in achieving both precision and recall.

On the other hand, the Bert-LSTM and Bert-GRU models display lower recall values, suggesting challenges in identifying positive instances. These models may be prone to false negatives, potentially missing positive sentiments in the CodeReview dataset. The Bert-CNN model, while achieving a high accuracy of 0.832, exhibits lower precision (0.723), indicating a higher rate of false positives. This trade-off between precision and recall is a critical consideration based on the specific requirements of the sentiment analysis task.

Interestingly, the proposed Fuzzy Ensemble model demonstrates notable improvements in performance on the CodeReview dataset. Despite the initial challenges posed by training models on a multi-class classification problem for a binary dataset, the Fuzzy Ensemble achieves an accuracy of 0.877, showcasing its adaptability and effectiveness in refining sentiment predictions. The precision (0.908) and recall (0.796) values suggest a well-balanced performance, resulting in a high F1-Score of 0.848. Additionally, the Cohen’s Kappa and ROC AUC values for the Fuzzy Ensemble align closely with those of the CQA-Bert model, indicating comparable overall effectiveness.

In summary, the CodeReview dataset’s binary nature poses challenges for models initially designed for multi-class classification. Despite this, the Fuzzy Ensemble model emerges as a promising solution, significantly enhancing sentiment classification performance and achieving results that are acceptable and competitive within the given context.

### 5.2 Fuzzy ensemble model comparison with existing state-of-the-art models

The Fuzzy Ensemble model is compared with the four state-of-the-art tools i.e. SentiStrength-SE [43], Senti4SD [22], SentiCR [38] and Generative Pre-Training Transformer (GPT) [44]. A brief overview of these tools is given in Table 8. GPT for domain-specific sentiment analysis was fine-tuned by authors for the comparison of the proposed fuzzy ensemble model with the GPT. GPT was implemented by using Huggingface library [45] in Colab platform [42] by using Python language. The hyper-parameters for GPT model implementation are given in Table 9. The values of these hyperparameters were set through Python code.

**Table 8:** Domain specific sentiment analysis tools for software engineering.

Tool	Dataset	Results
SentiStrength-SE [43]	Jira	73.85% precision and 85% recall
Senti4SD [22]	Stack Overflow	86% accuracy, 87% precision, and 86% recall
SentiCR [38]	CodeReview	83% accuracy, 67.8% precision, and 58.4% recall
GPT [44]	SentiSE	82% accuracy, 83% precision and 82% recall

**Table 9:** Parameters values for fine-tuning GPT.

Parameter	Value
Model	GPT2ForSequenceClassification
Batch Size	16
Max_Length	512
Learning Rate	1e-5
Epochs	5
Optimizer	AdamW

Performance evaluation was conducted across three diverse datasets, namely Jira, CodeReview, and Stack Overflow, chosen to comprehensively assess the sentiment analysis tools, as they were trained on these datasets. Precision, recall, and F1-Score metrics were employed for a rigorous evaluation, and the results are summarized in Table 10.

**Table 10:** Fuzzy ensemble model comparison with existing state-of-the-art models.

Dataset	Technique	Precision	Recall	F-Score
Jira	SentiStrength-SE	0.774	0.822	0.797
	Senti4SD	0.795	0.799	0.797
	SentiCR	**0.820**	**0.800**	**0.800**
	GPT	0.761	0.670	0.684
	Fuzzy Ensemble	**0.850**	**0.792**	**0.820**
CodeReview	SentiStrength-SE	0.669	**0.636**	**0.652**
	Senti4SD	**0.683**	0.609	0.644
	SentiCR	0.678	0.583	0.620
	GPT	0.836	0.795	0.813
	Fuzzy Ensemble	**0.908**	**0.796**	**0.848**
SO	SentiStrength-SE	0.800	0.800	0.800
	Senti4SD	0.860	0.870	0.860
	SentiCR	0.820	0.810	0.820
	GPT	0.824	0.810	0.810
	Fuzzy Ensemble	**0.898**	**0.890**	**0.894**

The results unequivocally establish the Fuzzy Ensemble model as a standout performer, surpassing the existing state-of-the-art sentiment analysis tools across all datasets. Notably, the precision of the Fuzzy Ensemble model significantly outstrips SentiStrength-SE across all datasets, underscoring its superior ability to accurately identify positive sentiments.

While SentiStrength-SE exhibits higher recall on the Jira dataset, the Fuzzy Ensemble model excels in achieving a more balanced and comprehensive performance, evident from its superior F1-Score across all datasets. This highlights the Fuzzy Ensemble’s effectiveness in correctly capturing positive sentiments while maintaining precision.

Comparing against Senti4SD, the Fuzzy Ensemble model demonstrates a substantial performance advantage, with markedly higher precision, recall, and F1-Score values on all datasets. This robust performance signifies the Fuzzy Ensemble’s efficacy in sentiment analysis within the software engineering domain.

In conclusion, the Fuzzy Ensemble model emerges as a powerful and versatile tool, consistently outperforming well-established sentiment analysis tools across diverse datasets. Its ability to achieve higher precision, recall, and F1-Score positions it as a valuable solution for sentiment classification tasks in the complex landscape of software engineering texts.

### 5.3 Consequences of fine-tuning the Large Language Model (LLM)

Fine-tuning the Large Language Model (LLM) can have a substantial impact on its performance and capabilities. One possible outcome is over-fitting, in which the model becomes highly specialized on the dataset on which it was trained. This specialization can limit the model’s ability to generalize to different tasks or datasets, lowering its overall efficacy. Another result of fine-tuning is catastrophic forgetting, which occurs when the LLM adapts to a new task and loses previously learned knowledge. This is especially relevant when the new task differs greatly from the original pre-training assignment, resulting in a decrease in performance [46]. Furthermore, fine-tuning might lead to a loss of generality because the model is optimized solely for the job for which it was fine-tuned. This can limit its performance across multiple tasks, reducing its overall value and versatility [47]. In this particular case, Bert Models are fine-tuned on the software engineering dataset. This will reduce the performance of the pre-trained Bert model on the on the generalized dataset. But on the other hand it will increase the performance of the Bert on the in predicting the software related tasks.

Despite these possible adverse effects, fine-tuning LLMs can provide benefits in certain situations. For example, aligning the model’s outputs with the preferences of various user groups might help stakeholders reach an agreement and consensus. Using a neurosymbolic architecture that blends the LLM’s flexibility with domain-specific knowledge can improve its adaptability and reasoning capabilities [46]. In this particular case, the fine-tuned Bert models is able to more accurately predict the software specific dataset thus it is overcoming the drawbacks of existing software specific sentiment analysis tools.

### 5.4 Practical application

One of the key advantages of the proposed module is its capacity to minimize manual work in categorizing user comments submitted on various community question and answer forums. Software engineers frequently spend time manually assessing and prioritizing user comments, which can be time-consuming and error-prone. The module automates this process, allowing software engineers to focus their time and skills on higher-value tasks like issue solving and innovation rather than wasting time on the labor-intensive effort of assessing user feedback.

Furthermore, by automating feedback analysis, proposed module allows for faster response times and more efficient decision-making. With automated analysis of user feedback, software development teams may quickly address issues, implement improvements, and prioritize feature enhancements, resulting in more responsive and user-centric software solutions. Overall, the practical deployment of our suggested module represents a big step forward in software engineering methods, providing a scalable and efficient solution to the issues of feedback analysis. We believe that by automating this crucial component of software development, our module will allow software engineers to provide higher-quality products more quickly and efficiently.

## 6 Conclusion and future work

Sentiment analysis, a challenging task for both machines and humans, has garnered substantial attention in the context of software engineering texts. As demonstrated by Pang and Lee [48], even human sentiment analysis accuracy hovers around 80%. In response to this challenge, our research undertook a large-scale evaluation of existing sentiment analysis tools for software engineering, coupled with the training of diverse classifiers to enhance accuracy. The extensive preprocessing phase was integral to refining the classifiers, ensuring their robust performance. The dataset, sourced from diverse software engineering interactions in SentiSE, served as a comprehensive benchmark for our evaluations. Notably, our proposed Fuzzy Ensemble model emerged as the epitome of accuracy across all datasets. Specifically, the Fuzzy Ensemble model achieved remarkable accuracy scores: 0.877 on the CodeReview dataset, 0.888 on the JavaLib dataset, 0.785 on the Jira dataset, and an outstanding 0.901 on the Stack Overflow dataset. These results substantiate the efficacy of the Fuzzy Ensemble model in accurately discerning sentiment within various software engineering contexts. Crucially, our evaluation positioned the Fuzzy Ensemble model as the superior performer compared to established state-of-the-art tools, including outperforming GPT on all datasets examined in this paper. This underscores the significance of our proposed model as a robust and versatile solution for sentiment analysis in the intricate landscape of software engineering texts. In conclusion, our research not only contributes valuable insights into the performance of sentiment analysis tools within the software engineering domain but also establishes the Fuzzy Ensemble model as a potent and accurate tool. The strides made in enhancing sentiment analysis accuracy hold promise for advancing the understanding of sentiment in software engineering, paving the way for more sophisticated and effective applications in the future.

In the future, a plan to extend domain-specific sentiment analysis tools to predict sentiments on aspect level is under consideration. The creation of a lexicon-based sentiment analysis tool to automatically extract lexicon from domain-specific datasets is also under process. The accuracy of the Fuzzy Ensemble model can be improved by using the balanced training dataset and a larger dataset for fine-tuning the Bert models. The results of the Fuzzy Ensemble model will be used to prioritize the software requirements and this model is part of the prioritization framework [49] that consists of Quality Assessment [50], sentiment detection, topic modeling and prioritization module [51].
